# Genetic Programming as Alternative for Predicting Development Effort of Individual Software Projects

**DOI:** 10.1371/journal.pone.0050531

**Published:** 2012-11-30

**Authors:** Arturo Chavoya, Cuauhtemoc Lopez-Martin, Irma R. Andalon-Garcia, M. E. Meda-Campaña

**Affiliations:** Department of Information Systems, CUCEA, Universidad de Guadalajara, Zapopan, Jalisco, Mexico; John Innes Centre, United Kingdom

## Abstract

Statistical and genetic programming techniques have been used to predict the software development effort of large software projects. In this paper, a genetic programming model was used for predicting the effort required in individually developed projects. Accuracy obtained from a genetic programming model was compared against one generated from the application of a statistical regression model. A sample of 219 projects developed by 71 practitioners was used for generating the two models, whereas another sample of 130 projects developed by 38 practitioners was used for validating them. The models used two kinds of lines of code as well as programming language experience as independent variables. Accuracy results from the model obtained with genetic programming suggest that it could be used to predict the software development effort of individual projects when these projects have been developed in a disciplined manner within a development-controlled environment.

## Introduction

The software process perspectives can be classified as follows [1]: organizations, teams and people. The performance of a software development organization is determined by the performance of its engineering teams, which in turn is determined by the performance of the team members, and the latter is, at least in part, determined by the practices these engineers follow in doing their work [2]. The levels of software engineering education and training of each developer can be applied both to small and large software projects [3]. Software development effort prediction is one of the three main practices used for training developers at a individual level; the other two are related to software defects and software size [2]. Software development prediction techniques could be classified into the following two general categories:

1. Expert judgment, which implies a lack of analytical argumentation and aims at deriving estimates based on the experience of experts on similar projects; this technique is based on a tacit (intuition-based) quantification step [4].
2. A model-based technique that is based on a deliberate (mechanical) quantification step; this category could be divided into the following two subcategories:
  - Models based on statistics whose general form is a linear or nonlinear statistical regression model [5].
  - Models based on machine learning techniques such as genetic programming, case-based reasoning, artificial neural networks, decision trees, Bayesian networks, support vector regression, genetic algorithms, and association rules. Among these methods, the application of genetic programming represents only the 3% of the techniques in the software effort prediction field [6].

Based on the assumption that no single technique is the best for all situations and that a careful comparison of the results of several approaches is more likely to produce realistic estimates [5], this study compares the accuracy of the following two models with each other: one model based on statistical regression, and a model based on genetic programming. The comparison against a statistical regression model is made because a regression analysis for selecting the significant variables should be done as the default model construction method [7] and because statistical regressions are the models most frequently compared with machine learning models [6].

Those two models were generated from data obtained from individually developed projects using practices of Personal Software Process (PSP). The use of PSP has proven its usefulness by thousands of practitioners when applied to individual projects [2]. The models of this research were generated from a dataset of 219 projects developed by 71 practitioners from the year 2005 to the year 2009. In order to validate these two models, they were applied to predict the effort of a new dataset consisting of 130 projects developed by 38 practitioners through the first semester of 2010.

In this work the accuracies of these two models are compared. This comparison is based upon the two following main stages when an estimation model is used [8]: (1) the model adequacy checking or model verification (estimation stage) must be determined, that is, whether the model is adequate to describe the observed (actual) data; if so then (2) the model is validated using new data (prediction stage).

The hypothesis to be investigated in this paper is the following:

H_1_: Effort prediction accuracy of a model based on genetic programming is statistically equal or better than that obtained by a multiple linear regression, when new and changed code, reused code, and programming language experience of developers data obtained from individually developed projects with personal practices are used as independent variables.

The foundation for predicting individual effort should be based on the assumption that unless software engineers have the capabilities provided by personal training, they cannot properly support their teams or consistently and reliably produce quality products. This assumption stems from the application of the Personal Software Process (PSP), whose practices and methods have been used by thousands of software engineers for delivering quality products on a predictable schedule [2]. This study is based upon the PSP practices described in the Methods section.

### 1.1 Genetic Programming

Genetic programming (GP) is a field of evolutionary computation that works by evolving a population of data structures that correspond to some form of computer programs [9]. These programs can typically be represented as trees varying in shape and size, where the internal nodes correspond to functions and the leaves represent terminals such as constant values and variable names. The trees can be implemented as the list-based structures known as S-expressions, with sublists representing subtrees.

**Figure 1:**
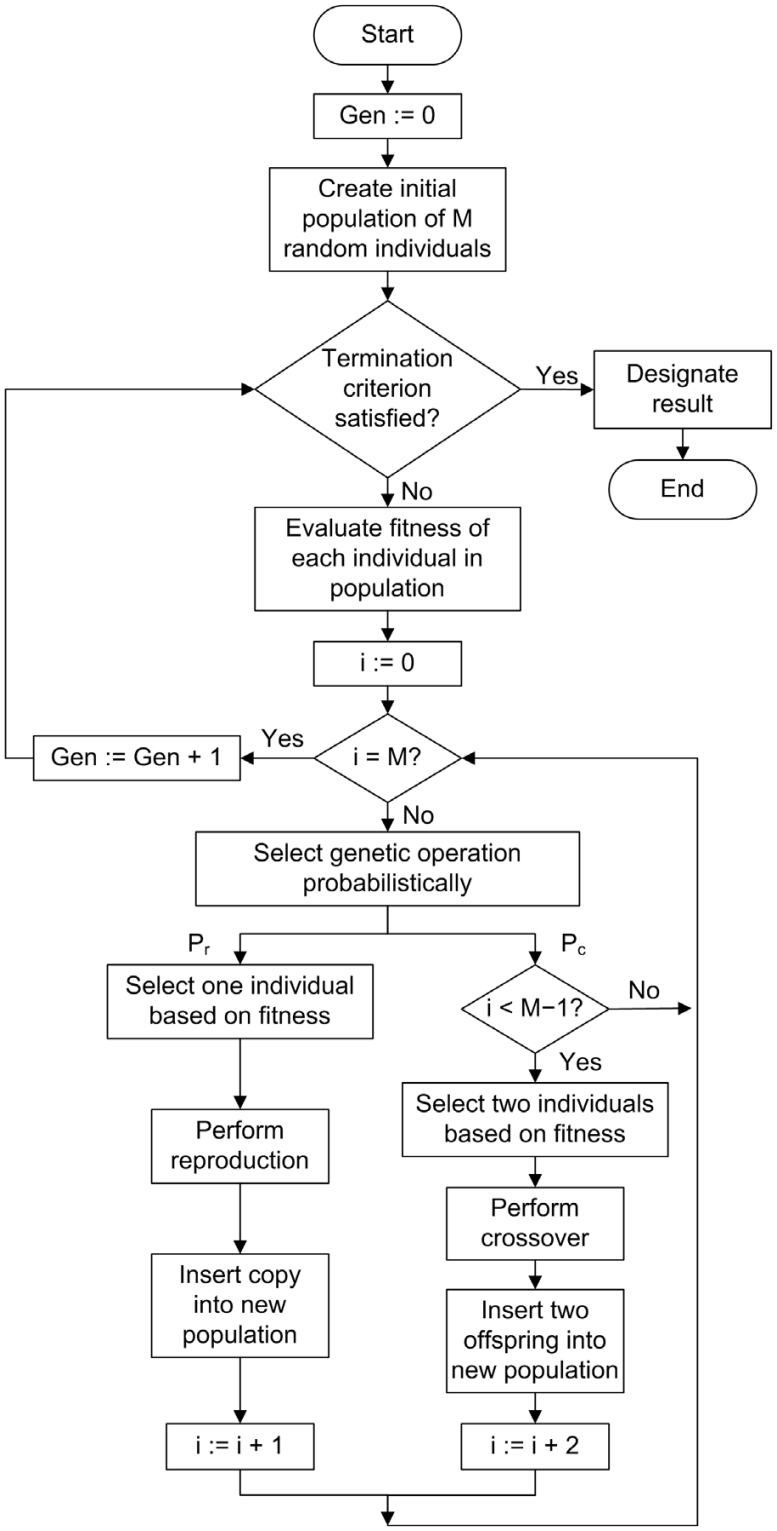
Flowchart followed by a typical implementation of the GP algorithm. Symbols are as follows: Gen = Generation counter; i: Individual counter; M: Population size; P_r_: Probability of reproduction; P_c_: Probability of crossover.

Figure 1 presents the flowchart followed by a typical implementation of the GP algorithm [9]. GP starts with a population of M randomly generated programs consisting of functions and terminals appropriate to the problem domain. If the termination criterion has not been satisfied, each program is then evaluated according to some fitness function that measures the ability of the program to solve a particular problem. The fitness function typically evaluates a problem against a number of different fitness cases and the final fitness value for the program is the sum or the average of the values of the individual fitness cases. GP normally works with a standardized fitness function in which lower non-negative values correspond to better values, usually with zero as the best value.

**Figure 2.Example:**
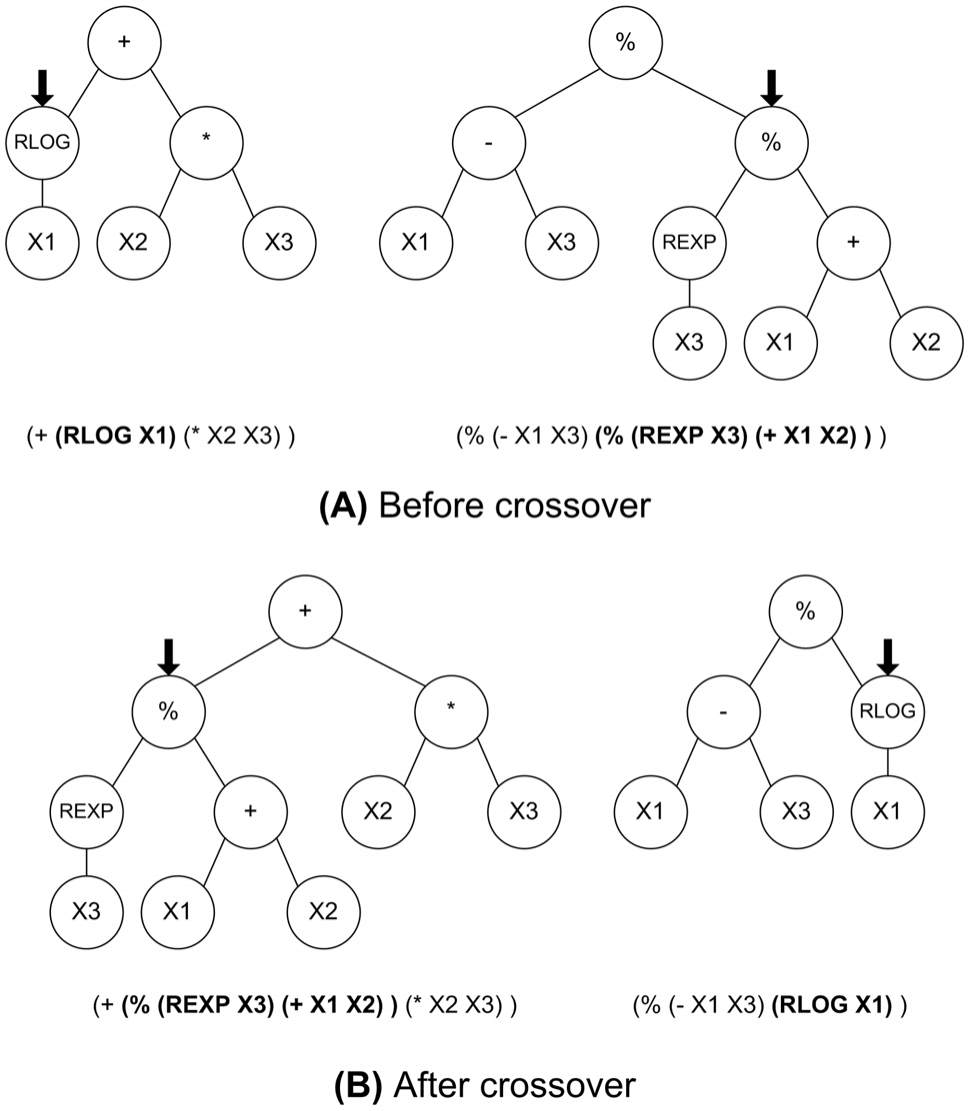
of crossover between two parental trees showing the corresponding S-expressions. A) Before crossover. B) After crossover. Arrows point at the root nodes of the subtrees that are to be exchanged. The subexpressions corresponding to the subtrees are shown in boldface.

After all programs in the population have been evaluated, a selection is made among the individuals in the population to produce the next generation. This selection is usually made proportionate to fitness so that programs with better fitness values have a higher probability of being selected. The Darwinian selection of the fittest individuals in the population is the biological basis upon which the various evolutionary computation paradigms are inspired. A number of operations can then be applied to selected individuals to provide for variability in the new generation. The reproduction operation consists of selecting a fixed percentage of individuals to pass unchanged to the next generation according to a certain probability of reproduction (P_r_). In the crossover operation, two individuals are selected according to a probability of crossover (P_c_) to function as parents to produce two offspring programs. In each of the parents a node in the corresponding trees is selected randomly to constitute a crossover point. The subtrees that have the selected nodes as roots are then exchanged generating two new individuals that are usually different from their parents. Figure 2A shows an example of two parental trees before crossover with the corresponding S-expression below each tree; arrows point at the root nodes of the subtrees chosen to be exchanged, with the corresponding subexpressions shown in boldface. Figure 2B presents the generated offspring trees resulting from the exchange of the subtrees whose root node is pointed at by an arrow. The exchange of subtrees corresponds to the exchange of the sublists shown in boldface below each tree.

A fixed portion of the next generation is produced using the crossover operation, having the possibility of forcing that a fixed percentage of the selected nodes correspond to functions, whereas the rest correspond to either functions or terminals. Unlike genetic algorithms, the mutation operation is normally not necessary in GP, as the crossover operation can provide for point mutation when two nodes corresponding to terminals are selected for exchange in the parents.

The process of evaluating, selecting and modifying individuals to produce a new generation is continued until a termination criterion is satisfied. The GP run usually terminates when either a predefined number of generations has been reached or a desired individual has been found.

### 1.2 Measurement of Software Projects

Source lines of code (LOC) represent one of the two main measures for sizing software projects [10]. There are two measures of source code size: physical source lines and logical source statements. The count of physical lines gives the size in terms of the physical length of the code as it appears when printed [11]. In this study, two of the independent variables are New and Changed (N&C) as well as Reused code and they were considered as physical LOC. N&C is composed of added and modified code; the added code is the LOC written during the current programming process, whereas the modified code is the LOC changed in the base program when modifying a previously developed program. The base program is the total LOC of the previous project, whereas the reused code is the LOC of previously developed programs that are used without any modification [12]. A coding standard should establish a consistent set of coding practices that is used as a criterion when judging the quality of the produced code [12]. Hence, it is necessary to always use the same coding and counting standards. The software projects of this study followed those two guidelines.

After product size, people factors –such as experience on applications–, platforms, languages and tools have the strongest influence in determining the amount of effort required to develop a software product [5]. Programming language experience is used in this study as a third independent variable, which was measured in months as units. Development effort was measured in minutes.

### 1.3 Accuracy Criterion

Common criteria for the evaluation of prediction models have been the Magnitude of Relative Error (MRE) and the Magnitude of Error Relative to the estimate or MER [13]. MRE and MER are defined as follows
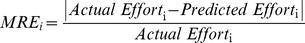


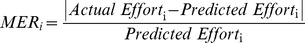



The MRE and MER values are calculated for each observation *i* whose effort is predicted. The aggregation of MRE and of MER over multiple observations (*N*) can be achieved through their mean (MMRE and MMER) as follows:
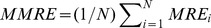


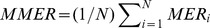



Intuitively, MER seems preferable to MRE since MER measures the error relative to the estimate. Results of MMER in [13] had better results than MMRE; this fact is the reason for using MMER as our main criterion.

The accuracy of a prediction technique is inversely proportional to its MMRE or MMER. In a number of papers, an MMRE≤0.25 has been considered as acceptable, however, authors who have proposed this value [14] neither present any reference to studies nor any argumentation providing evidence [15]. On the other hand, a reference for an acceptable value of MMER has not been reported.

### 1.4 Related Work

Genetic programming (GP) has already been applied to large projects [16]. However, we did not find any study related to its application for predicting the software development effort of individually developed projects in laboratory learning environments, and whose independent variables had been related to the three ones described in Section 1.2. Some of the methods reported in previous publications resemble the approach taken in the present work in which a mathematical model that best fits the data is searched. The main difference of the present work with previous reports lies in the genetic programming parameters they used and the data on which they applied the genetic programming algorithm. A GP algorithm was implemented in [17] having a population size of 1000 individuals reproducing for 500 generations during 10 runs only. They used a dataset of 81 software projects that a Canadian software company developed in the late 1980s. They suggested that the GP approach needed further study to fully exploit its advantages. On the other hand, GP was used in [18] with the goal of comparing the use of public datasets against company-specific ones. The techniques they used (genetic programming, artificial neural networks and multiple linear regression) were slightly more accurate with the company-specific database than with publicly available datasets. They used the same genetic programming parameters as in [17]. They concluded that companies should base effort estimates on in-house data rather than on public domain data. GP was compared in [19] against artificial neural networks and multiple linear regression using a number of publicly available datasets. Using less individuals in the GP population (from 25 to 50) than normally employed in the typical implementation of the algorithm (several hundred), they found that although GP was better at effort prediction than neural networks and multiple linear regression with some datasets, in general none of the techniques they tested rendered an appropriate effort estimation model. These authors concluded that the datasets used to build a prediction model had a great influence in the ability of the model to provide adequate effort estimation. A different approach was used in [20] with GP; instead of finding the mathematical model that best fitted the data, they developed a grammar-based technique they called Grammar Guided Genetic Programming (GGGP) and compared it against simple linear regression. They used the data of 423 software development projects from a public repository and randomly divided them into a training set of 211 projects and a test set of 212 projects. The results obtained using the GGGP technique were not very encouraging, as the effort prediction they found was not very accurate.

Table 1 presents the accuracy by model from the mentioned studies. These four analyses used more than one accuracy criterion; the MMRE was always amongst them, whereas none of them used MMER. The use of the “best” MMRE in Table 1 is because the authors of [19] used five datasets, where one of them was an approximation of the actual one; in contrast, Table 1 presents only the best MMRE of the others four datasets. In Table 1, GP had the best MMRE in three of the four studies, although the best of them had only a value of 0.34.

**Table 1:** MMER comparison.

Study	SS for GM	SS for VM	Total	Best MMRE	
				SR	GP
Burguess et al. [17]	63	18	81	0.46	0.45
Lefley et al. [18]	149	15	154	0.45	0.38
Shan et al. [20]	211	212	423	1.94	1.91
Dolado et al. [20]	33	15	48	0.30	0.34

SS: Sample size measured in number of projects; GM: Generation of Models; VM: Validation of models; SR: Statistical regression; GP: Genetic programming.

Finally, GP was also applied in [21] for predicting the effort of large projects, and their results showed that GP was better than case-based reasoning and comparable with statistical regression.

## Methods

Experiments for this study were done within a controlled environment having the following characteristics:

1. All of the developers were experienced and were working for some software development company. However, none of them had previously taken a course related to personal practices for developing software at the individual level.
2. All developers were studying a graduate program related to computer science.
3. The kind of the developed projects had a similar complexity as those suggested in [12]. From a set of 18 projects, a subset of seven projects was randomly assigned to each of the practitioners. Description of these 18 projects is presented in [22].
4. Each developer wrote seven project assignments. Only the last four of the assignments of each developer were selected for this study. The first three projects were not considered because they had differences in their process phases and in their logs, whereas the last four projects were based on the same logs and had the following phases: plan, design, design review, code, code review, compile, testing and postmortem.
5. Each developer selected his/her own imperative programming language whose coding standard had the following characteristics: each compiler directive, variable declaration, constant definition, delimiter, assign sentence, as well as flow control statement was written in one line of code.
6. Since a coding standard establishes a consistent set of coding practices that is used as a criterion for judging the quality of the produced code [12], the same coding and counting standards were used in all projects. The projects developed during this study followed these guidelines. All projects adhered to the counting standard shown in Table 2.
7. Developers had already received at least a formal course on the object oriented programming language that they selected to be used through the assignments, and they had good programming experience in the chosen language. The sample for this study only involved developers whose projects were coded in C++ or JAVA. Comparisons for new and changed (N&C) and effort (shown in Appendix S1) were done between the two languages. The p-value for N&C was equal to 0.90, whereas that for effort was 0.79, i.e., for these two variables there was not a statistically significant difference between the two programming languages at the 95.0% confidence level. The assumptions of residuals were analyzed and achieved (independent samples, equal standard deviations, and normal populations).
8. Because this study was an experiment with the aim of reducing bias, we did not inform the developers about our experimental goal.
9. Developers filled out a spreadsheet for each project and submitted it electronically for examination.
10. Each PSP course was taught to no more than fifteen developers.
11. Developers were constantly supervised and advised about their process.
12. The code written in each project was designed by the developers to be reused in subsequent projects.
13. Data used in this study are from those practitioners whose data for all seven exercises were correct, complete, and consistent.

**Table 2:** Counting standard.

Count type	Type
Physical/logical	Physical
**Statement type**	**Included**
Executable	Yes
Non-executable	
Declarations	Yes (one per text line)
Compiler directives	Yes (one per text line)
Comments	No
Blank lines	No
**Delimiters**	
* {* and*}*	Yes

The following two subsections describe how the statistical regression and genetic programming models were generated from the actual data presented in Appendix S1. These models were generated from a dataset of 219 projects developed by 71 practitioners from the year 2005 to the year 2009.

### 2.1 Multiple Linear Regression

The following multiple linear regression equation considering New and Changed (*N*&*C*), *Reused* code and Programming Language Experience (*PLE*) was generated:;
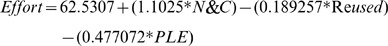



It has been suggested that an acceptable value for a coefficient of determination is r^2^≥0.5 when it comes to software development effort estimation [12]. This equation had an r^2^ = 0.51. An analysis of variance (ANOVA) for this equation (Table 3) shows a statistically significant relationship between the variables at a 99% confidence level. In order to determine whether the model could be simplified, a parameter analysis of the multiple linear regression was done. Table 4 shows the results for this analysis; the highest p-value on the independent variables is 0.0027, corresponding to reused code. Since this p-value is less than 0.05, reused code is statistically significant at a 95% confidence level. Consequently, the independent variable of reused code was not removed. Hence, this variable had to be considered for its evaluation.

**Table 3:** ANOVA of Multiple Linear Regression Analysis.

Source	Sum of squares	Degrees of freedom	Mean square	F-ratio	p-value
Model	124825	3	41608.5	74.99	0.000
Residual	119299	215	554.88		
Total	244125	218			

**Table 4:** Individual analysis of parameters.

Parameter	Estimate	Standard error	t statistic	p-value
Constant	62.5307	4.68365	13.3509	0.0000
N&C	1.1025	0.0766589	14.3819	0.0000
Reused	−0.189257	0.0623356	−3.0361	0.0027
PLE	−0.477072	0.102896	−4.63644	0.0000

### 2.2 Genetic Programming Model

A LISP implementation of the GP algorithm was used for generating a model to predict software development effort. The following standard parameters were used on all runs [9]: the initial population consisted of 500 S-expressions randomly generated using the ramped half-and-half generative method. In this method, an equal number of trees are created with a depth that ranges from 2 to the maximum allowed depth (6 in this work) for new individuals. For each depth, half of the programs correspond to full trees, and the other half consist of growing trees of variable shape. Maximum depth for individuals after the application of the crossover operation was 17. Reproduction rate was 0.1, whereas crossover rate was 0.7 for function nodes and 0.2 for any node. Finally, each GP run was allowed to evolve for 50 generations and the individual with the best fitness value was selected.

The set of terminals was defined by the three independent variables *X1, X2* and *X3* corresponding to New & Changed LOC, Reused LOC, and Programming Language Experience in months, respectively. Additionally, terminals also consisted of floating-point constants randomly generated from the range [−5, 5).

The set of functions consisted of the arithmetic operators for addition (+), subtraction (−) and multiplication (*), along with the following protected functions shown in prefix notation. To avoid division by zero, the protected division % was defined as follows:
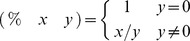



To account for non-positive variable values, the protected logarithmic function *RLOG* was defined as:
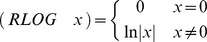



Finally, the protected exponential function *REXP* was defined as:
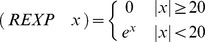



where the boundary value 20 was arbitrarily chosen to avoid over- and underflows during evaluation.

Since the standardized fitness function *f* is required to consist of non-negative values, with zero as the best match, this function was defined as
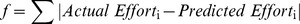



The MMER value was not considered an appropriate fitness measure, as the denominator in the MER formula can give negative values if the estimated effort is negative itself.

## Results and Discussion

### 3.1 Genetic Programming Experiments

One hundred experiments each consisting of 1000 GP runs were made. From each experiment, the run with the highest fitness value (lowest *f* value) was selected and finally an individual program from all experiments was selected according to how well it predicted software development effort on both the verification and validation datasets.

**Figure 3:**
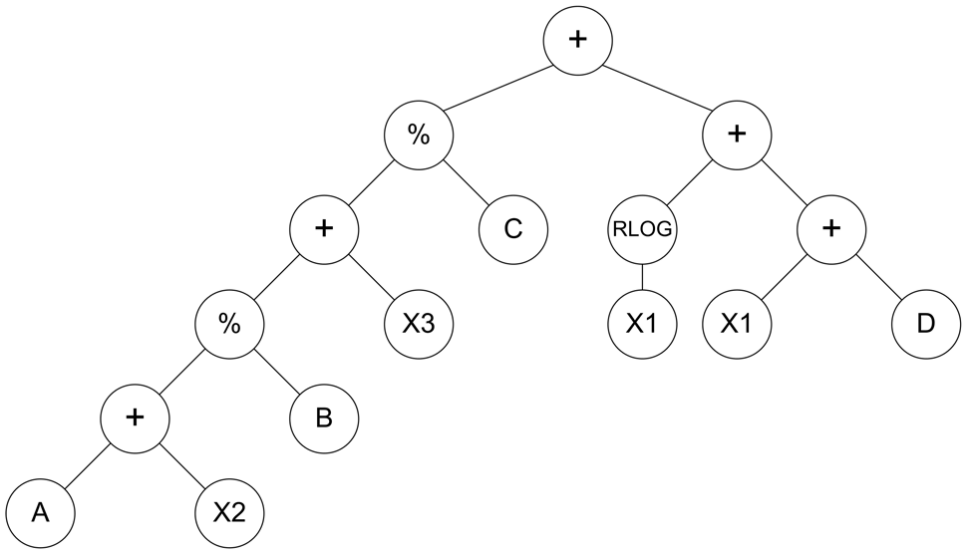
Tree representation of the LISP program corresponding to the best solution. Variables are as follows: X1 =  New & Changed LOC, X2 =  Reused LOC, and X3 =  Programming Language Experience in months. Constant values are as follows: A = 1.1211268, B = 1.5219285, C = −3.4280592, and D = 59.091568.

The selected program from the 100,000 runs is presented next in LISP notation:
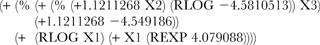



After evaluation of constant subexpressions, the following equivalent program was obtained:
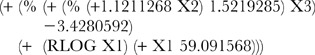



The tree representation of this program is shown in Figure 3.

### 3.2 Verification of Models

Once the multiple linear regression equation and the genetic programming algorithm (presented in Sections 2.1 and 2.2, respectively) were applied to the original dataset (see Appendix S1), a MER by software project as well as a MMER by model was then calculated. Table 5 presents the MMER by model once they were generated and applied to the set of 219 projects.

**Table 5:** MMER by model obtained from verification stage.

Model	MMER
Multiple linear regression	0.25
Genetic programming	0.25

The ANOVA for MER of the projects (Table 6) shows that there is not a statistically significant difference amongst the accuracy of prediction for the two models at the 95.0% confidence level.

**Table 6:** MER ANOVA (verification of models).

Source	Sum of squares	Degrees of freedom	Mean square	F-ratio	p-value
Between groups	0.00029	1	0.00029	0.01	0.9176
Within groups	12.0371	436	0.02760		
Total	12.0374	437			

**Figure 4:**
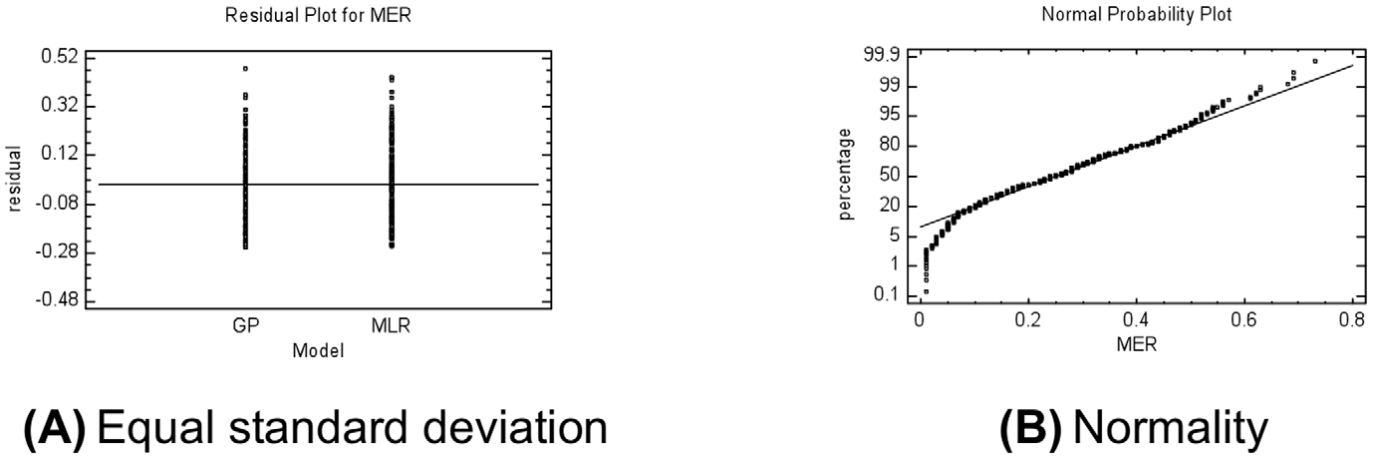
Plots of MER ANOVA (verification stage). A) The residuals should fall roughly in a horizontal band centered and symmetrical about the horizontal axis in the plot; GP: Genetic programming, MLR: Multiple linear regression. B) A normal probability plot of the residuals should be roughly linear.

The following three assumptions of residuals for MER ANOVA were analyzed:

1. Independent samples: in this study, practitioners separately developed each software project; hence the data are independent.
2. Equal standard deviations: In a plot of this kind the residuals should fall roughly in a horizontal band centered and symmetrical about the horizontal axis (as shown in Figure 4A), and
3. Normal populations: A normal probability plot of the residuals (the Shapiro-Wilk test) should be roughly linear (as shown in Figure 4B).

### 3.3 Validation of Models

**Figure 5:**
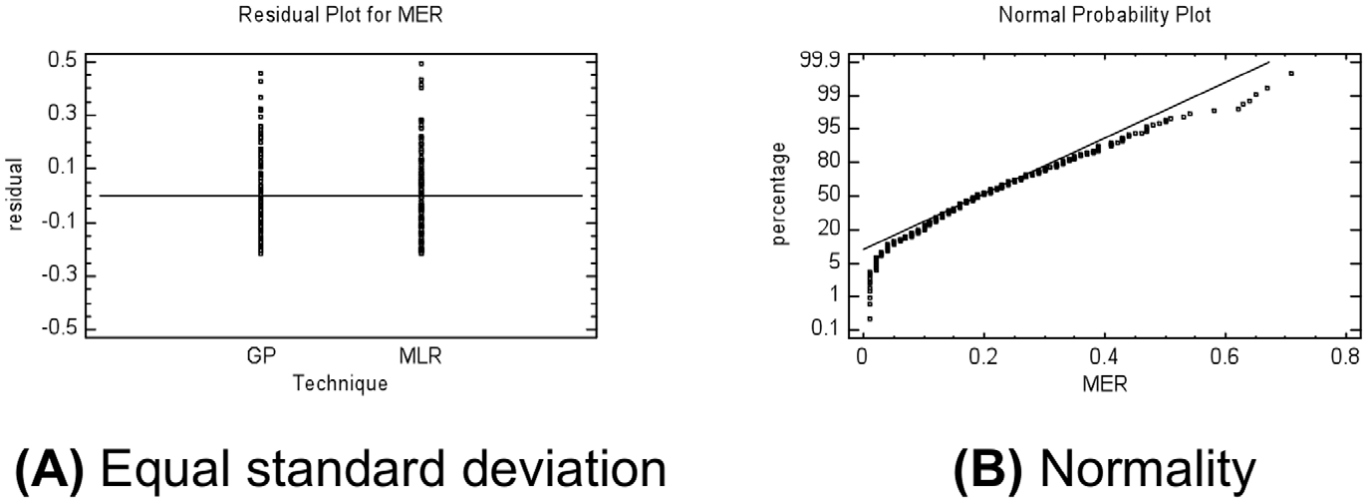
Plots of MER ANOVA (validation stage). A) The residuals should fall roughly in a horizontal band centered and symmetrical about the horizontal axis in the plot; GP: Genetic programming, MLR: Multiple linear regression. B) A normal probability plot of the residuals should be roughly linear.

A second group involving thirty-eight practitioners developed 130 software projects through the first semester of 2010 based on the same characteristics of the experiment described in the Methods section. This new sample was used for validating the two models. In Appendix S2, actual data from these projects are presented. The MMER results by model are shown in Table 7. In accordance with the ANOVA for MER models (Table 8), there is not a statistically significant difference between the prediction accuracy for the two models at the 95.0% confidence level. Figures 5A and 5B show respectively that residuals related to equal standard deviations as well as with normality data of this ANOVA were met.

**Table 7:** MMER by model obtained from the validation stage.

Model	MMER
Multiple linear regression	0.22
Genetic programming	0.21

**Table 8:** MER ANOVA (validation of models).

Source	Sum of squares	Degrees of freedom	Mean square	F-ratio	p-value
Between groups	0.0006	1	0.00067	0.03	0.8631
Within groups	5.8732	258	0.02276		
Total	5.8738	259			

### Conclusions

The levels of software engineering education and training can be classified into small and large projects. This research focused its interests on personal training based on individually developed projects and using PSP whose practices and methods are used by thousands of developers for delivering quality products on predictable schedules.

Estimation and prediction of the software development effort were done based on an accuracy comparison between the models obtained with multiple linear regression (MLR) and genetic programming.

The models used in this research were generated from a dataset of 219 projects developed by 71 practitioners from the year 2005 to the year 2009 and then these two models were applied for predicting the effort of a new dataset that consisted of 130 projects developed by 38 practitioners through the first semester of 2010. Three independent variables were used for generating the two models; two of those variables were related to source code size and the third one was related to the developers’ programming language experience.

The accepted hypothesis was the following:

H_1_: Effort prediction accuracy of a model based on genetic programming is statistically equal than that obtained by a multiple linear regression, when new and changed code, reused code, and programming language experience of developers data obtained from individually developed projects with personal practices are used as independent variables.

This hypothesis suggests that genetic programming could be used for predicting the development effort of individual projects when they have been developed using personal practices.

In this study, the GP technique used was proposed based on the following reasons:

1. Software organizations involve teams of developers and the performance of these teams is determined in part by the performance of individuals. One of the main activities of a developer is the prediction of his/her effort for developing small projects, which will be part of a larger system. Most previous studies involving GP have applied it for the development effort prediction of large projects, whereas its application to individual software projects has been limited.
2. It has been suggested that more than one technique should be used for predicting software development effort [5].
3. GP is able to model non-linear behaviors, which are common when independent variables are correlated to development effort of software projects [23].

Although machine learning techniques have been receiving increasing attention in software development effort prediction research, empirical studies on the application of GP are still scarce [6]. Furthermore, previous studies have been inconclusive in judging whether or not GP is an effective technique for software development effort prediction. The reasons for this are: (1) while GP optimizes one accuracy measure, it degrades others, and (2) the experimental procedures used among previous studies varied, with different strategies used for sampling the training and the testing sets [16].

In addition to the results obtained in [16], the use of GP as an alternative could also be supported by the following reasons obtained from a study where GP was compared to other machine learning techniques, namely neural networks (NN), support vector regression (SVR), case-based reasoning (CBR), decision trees (DT), and Bayesian networks (BN) [6]:

1. GP showed acceptable prediction accuracy: it was only outperformed by NN and SVR; it was equal in prediction accuracy to CBR and DT, and it was better than BN.
2. GP had lesser variation in its prediction accuracy than NN, SVR, CBR, DT and BN.

The small number of studies where comparisons between GP and regression models have been done limits the generalization of the comparison results [6] [16]; hence, this study contributes to alleviate this problem.

Future research involves a predictive accuracy comparison applying genetic algorithms as well as other optimization techniques for data obtained from large systems built by teams of developers.

## Supporting Information

Appendix S1 **Data of projects for generating and verifying models.**
(DOC)

Appendix S2 **Data of projects for validating models.**
(DOC)
