# Supplementary material for: Genetic Programming as Alternative for Predicting Development Effort of Individual Software Projects
Source: PLoS One. 2012 Nov 30;7(11):e50531. doi: 10.1371/journal.pone.0050531 (PMC3511534; doi:10.1371/journal.pone.0050531)
Supplement: Appendix S1 — Data of projects for generating and verifying models. (DOC) [file pone.0050531.s001.doc]

**Appendix A.** Data of projects for generating and verifying models.

| PLE | R | N&C | E | MLR | GP | PLE | R | N&C | E | MLR | GP | PLE | R | N&C | E | MLR | GP |
| --- | --- | --- | --- | --- | --- | --- | --- | --- | --- | --- | --- | --- | --- | --- | --- | --- | --- |
| 24 | 21 | 17 | 65 | 65.85 | 67.68 | 48 | 47 | 19 | 55 | 51.68 | 57.81 | 12 | 108 | 18 | 34 | 56.21 | 55.57 |
| 24 | 24 | 36 | 100 | 86.23 | 86.86 | 48 | 31 | 21 | 43 | 56.92 | 62.98 | 12 | 37 | 35 | 42 | 88.39 | 86.84 |
| 24 | 11 | 42 | 100 | 95.30 | 95.50 | 48 | 12 | 27 | 55 | 67.13 | 72.87 | 12 | 40 | 36 | 81 | 88.93 | 87.29 |
| 14 | 8 | 95 | 171 | 159.08 | 152.81 | 48 | 49 | 31 | 54 | 64.54 | 69.92 | 12 | 50 | 13 | 51 | 61.68 | 61.36 |
| 14 | 30 | 97 | 131 | 157.12 | 150.62 | 48 | 42 | 11 | 23 | 43.81 | 50.22 | 12 | 48 | 13 | 58 | 62.05 | 61.74 |
| 24 | 78 | 34 | 43 | 73.80 | 74.45 | 48 | 8 | 15 | 42 | 54.65 | 61.05 | 12 | 43 | 17 | 89 | 67.41 | 66.97 |
| 24 | 38 | 35 | 52 | 82.48 | 83.15 | 48 | 33 | 24 | 51 | 59.85 | 65.73 | 12 | 25 | 30 | 71 | 85.15 | 83.99 |
| 24 | 80 | 50 | 52 | 91.07 | 90.45 | 48 | 5 | 44 | 97 | 87.19 | 91.70 | 8 | 94 | 54 | 100 | 100.46 | 96.51 |
| 24 | 52 | 54 | 56 | 100.77 | 99.90 | 48 | 46 | 75 | 115 | 113.61 | 115.38 | 12 | 64 | 8 | 53 | 53.51 | 53.19 |
| 12 | 33 | 11 | 44 | 62.69 | 62.45 | 48 | 17 | 100 | 153 | 146.66 | 146.22 | 12 | 30 | 22 | 84 | 75.38 | 74.72 |
| 12 | 27 | 14 | 32 | 67.13 | 66.84 | 48 | 111 | 115 | 144 | 145.41 | 143.34 | 12 | 40 | 29 | 79 | 81.21 | 80.08 |
| 12 | 8 | 25 | 50 | 82.85 | 82.06 | 36 | 33 | 15 | 38 | 55.65 | 59.76 | 12 | 19 | 40 | 68 | 97.31 | 95.42 |
| 36 | 42 | 10 | 35 | 48.43 | 52.63 | 36 | 46 | 37 | 50 | 77.44 | 80.17 | 12 | 71 | 16 | 77 | 61.01 | 60.54 |
| 36 | 25 | 12 | 38 | 53.85 | 58.07 | 36 | 42 | 54 | 100 | 96.94 | 98.31 | 12 | 71 | 37 | 74 | 84.16 | 82.38 |
| 36 | 22 | 23 | 54 | 66.55 | 70.29 | 48 | 7 | 63 | 70 | 107.76 | 110.68 | 12 | 31 | 40 | 122 | 95.04 | 93.12 |
| 36 | 33 | 31 | 61 | 73.29 | 76.48 | 48 | 43 | 79 | 133 | 118.59 | 120.00 | 12 | 39 | 71 | 177 | 127.70 | 123.16 |
| 12 | 44 | 15 | 41 | 65.02 | 64.65 | 48 | 46 | 10 | 30 | 41.95 | 48.36 | 36 | 70 | 19 | 35 | 53.06 | 56.90 |
| 12 | 36 | 21 | 32 | 73.15 | 72.52 | 48 | 57 | 11 | 34 | 40.97 | 47.35 | 36 | 74 | 32 | 82 | 66.63 | 69.66 |
| 12 | 23 | 22 | 41 | 76.71 | 76.06 | 48 | 40 | 12 | 39 | 45.29 | 51.69 | 36 | 21 | 66 | 84 | 114.15 | 114.54 |
| 12 | 38 | 24 | 39 | 76.07 | 75.27 | 48 | 52 | 12 | 62 | 43.02 | 49.39 | 6 | 104 | 29 | 85 | 71.96 | 69.56 |
| 60 | 25 | 40 | 112 | 73.27 | 80.27 | 3 | 51 | 12 | 58 | 64.68 | 62.71 | 6 | 9 | 58 | 99 | 121.91 | 117.46 |
| 36 | 31 | 14 | 79 | 54.92 | 59.07 | 3 | 65 | 16 | 59 | 66.44 | 64.32 | 6 | 36 | 59 | 85 | 117.90 | 113.30 |
| 36 | 33 | 31 | 85 | 73.29 | 76.48 | 3 | 18 | 38 | 145 | 99.59 | 96.19 | 6 | 6 | 67 | 97 | 132.40 | 127.18 |
| 36 | 31 | 35 | 107 | 78.08 | 80.99 | 24 | 49 | 10 | 39 | 52.83 | 54.79 | 60 | 15 | 35 | 117 | 69.66 | 77.05 |
| 24 | 40 | 18 | 70 | 63.36 | 65.10 | 24 | 36 | 24 | 92 | 70.73 | 72.15 | 60 | 30 | 44 | 112 | 76.74 | 83.41 |
| 24 | 33 | 18 | 97 | 64.68 | 66.44 | 24 | 22 | 29 | 98 | 78.89 | 80.03 | 36 | 100 | 41 | 58 | 71.63 | 73.92 |
| 48 | 10 | 46 | 83 | 88.45 | 92.79 | 24 | 16 | 60 | 156 | 114.20 | 112.90 | 36 | 62 | 44 | 74 | 82.13 | 84.28 |
| 24 | 32 | 81 | 155 | 134.33 | 131.14 | 6 | 49 | 16 | 115 | 68.03 | 66.51 | 36 | 82 | 49 | 48 | 83.86 | 85.55 |
| 24 | 100 | 119 | 168 | 163.35 | 156.49 | 6 | 54 | 31 | 90 | 83.63 | 81.21 | 36 | 86 | 95 | 111 | 133.82 | 131.45 |
| 24 | 38 | 15 | 30 | 60.43 | 62.30 | 24 | 14 | 15 | 63 | 64.97 | 66.90 | 24 | 48 | 8 | 20 | 50.82 | 52.75 |
| 24 | 44 | 18 | 32 | 62.60 | 64.33 | 24 | 14 | 16 | 40 | 66.07 | 67.96 | 24 | 22 | 12 | 53 | 60.15 | 62.14 |
| 24 | 12 | 37 | 50 | 89.60 | 90.19 | 24 | 10 | 24 | 113 | 75.65 | 77.14 | 24 | 9 | 23 | 38 | 74.74 | 76.29 |
| 60 | 27 | 12 | 57 | 42.03 | 50.68 | 24 | 5 | 26 | 60 | 78.80 | 80.18 | 24 | 15 | 31 | 45 | 82.42 | 83.43 |
| 60 | 35 | 20 | 65 | 49.33 | 57.66 | 5 | 33 | 15 | 90 | 70.44 | 68.80 | 6 | 86 | 18 | 87 | 63.24 | 61.53 |
| 60 | 9 | 22 | 42 | 56.46 | 64.74 | 5 | 15 | 39 | 135 | 100.30 | 97.21 | 6 | 55 | 34 | 116 | 86.74 | 84.11 |
| 60 | 8 | 29 | 49 | 64.36 | 72.21 | 5 | 10 | 40 | 120 | 102.35 | 99.19 | 6 | 41 | 48 | 120 | 104.83 | 101.14 |
| 12 | 30 | 75 | 104 | 133.82 | 128.94 | 5 | 12 | 40 | 131 | 101.97 | 98.81 | 36 | 64 | 23 | 90 | 58.60 | 62.24 |
| 12 | 63 | 39 | 98 | 87.88 | 85.96 | 18 | 53 | 13 | 83 | 58.25 | 59.03 | 36 | 64 | 49 | 129 | 87.27 | 89.00 |
| 4 | 15 | 20 | 114 | 79.83 | 77.83 | 18 | 22 | 31 | 88 | 83.96 | 83.84 | 36 | 19 | 49 | 142 | 95.78 | 97.63 |
| 4 | 35 | 41 | 121 | 99.20 | 95.71 | 6 | 38 | 23 | 54 | 77.83 | 75.98 | 24 | 98 | 30 | 107 | 65.61 | 66.49 |
| 4 | 69 | 81 | 129 | 136.87 | 129.88 | 6 | 28 | 25 | 91 | 81.93 | 79.98 | 24 | 78 | 52 | 115 | 93.65 | 92.88 |
| 24 | 23 | 70 | 133 | 123.90 | 121.72 | 6 | 39 | 27 | 35 | 82.05 | 79.95 | 24 | 52 | 59 | 111 | 106.29 | 104.99 |
| 36 | 33 | 11 | 74 | 51.24 | 55.45 | 6 | 12 | 41 | 71 | 102.60 | 99.54 | 24 | 10 | 72 | 132 | 128.57 | 126.24 |
| 36 | 56 | 19 | 59 | 55.71 | 59.59 | 48 | 39 | 18 | 60 | 52.10 | 58.29 | 6 | 50 | 12 | 67 | 63.44 | 62.03 |
| 36 | 11 | 23 | 71 | 68.63 | 72.40 | 48 | 8 | 43 | 119 | 85.52 | 90.10 | 6 | 57 | 15 | 56 | 65.42 | 63.91 |
| 36 | 40 | 26 | 102 | 66.45 | 69.97 | 48 | 12 | 56 | 87 | 99.10 | 102.60 | 6 | 15 | 18 | 83 | 76.67 | 75.14 |

PLE: Programming language experience (months), R: Reused code, N&C: New and changed code, E: Effort (minutes), MLR: Effort predicted from the multiple linear regression model, GP: Effort predicted from genetic programming model.

**Appendix A (continued).** Data of projects for generating and verifying models.

| PLE | R | N&C | E | MLR | GP | PLE | R | N&C | E | MLR | GP |
| --- | --- | --- | --- | --- | --- | --- | --- | --- | --- | --- | --- |
| 6 | 40 | 30 | 106 | 85.17 | 82.86 | 12 | 81 | 68 | 115 | 116.45 | 112.07 |
| 6 | 55 | 17 | 86 | 68.00 | 66.42 | 12 | 23 | 18 | 87 | 72.30 | 71.86 |
| 24 | 54 | 18 | 57 | 60.71 | 62.42 | 12 | 52 | 24 | 112 | 73.42 | 72.59 |
| 24 | 70 | 32 | 70 | 73.11 | 73.92 | 12 | 77 | 31 | 57 | 76.41 | 75.05 |
| 24 | 10 | 58 | 105 | 113.13 | 112.02 | 12 | 70 | 52 | 130 | 100.89 | 97.91 |
| 24 | 55 | 77 | 94 | 125.56 | 122.68 | 36 | 77 | 25 | 61 | 58.35 | 61.84 |
| 12 | 99 | 28 | 46 | 68.94 | 67.73 | 36 | 40 | 35 | 81 | 76.37 | 79.26 |
| 12 | 84 | 29 | 72 | 72.88 | 71.64 | 36 | 58 | 35 | 100 | 72.97 | 75.81 |
| 12 | 69 | 40 | 92 | 87.85 | 85.84 | 12 | 47 | 12 | 92 | 61.14 | 60.85 |
| 12 | 60 | 45 | 88 | 95.06 | 92.68 | 12 | 5 | 32 | 98 | 91.14 | 89.88 |
| 12 | 100 | 46 | 70 | 88.60 | 86.04 | 12 | 10 | 36 | 82 | 94.60 | 93.04 |
| 12 | 35 | 67 | 144 | 124.05 | 119.87 | 12 | 19 | 45 | 128 | 102.82 | 100.54 |
| 36 | 32 | 18 | 42 | 59.14 | 63.13 | 36 | 100 | 15 | 29 | 42.97 | 46.92 |
| 36 | 15 | 27 | 60 | 72.28 | 75.80 | 36 | 53 | 21 | 45 | 58.48 | 62.26 |
| 36 | 32 | 30 | 39 | 72.37 | 75.64 | 36 | 88 | 38 | 32 | 70.60 | 73.15 |
| 36 | 5 | 40 | 59 | 88.51 | 91.11 | 12 | 57 | 13 | 76 | 60.35 | 60.02 |
| 12 | 97 | 12 | 33 | 51.68 | 51.27 | 12 | 35 | 22 | 107 | 74.44 | 73.76 |
| 12 | 39 | 37 | 117 | 90.22 | 88.51 | 12 | 24 | 33 | 126 | 88.65 | 87.27 |
| 12 | 44 | 82 | 155 | 138.88 | 133.35 | 48 | 30 | 8 | 38 | 42.77 | 49.20 |
| 36 | 111 | 41 | 92 | 69.55 | 71.81 | 48 | 34 | 22 | 75 | 57.45 | 63.45 |
| 36 | 82 | 77 | 162 | 114.73 | 114.00 | 48 | 8 | 23 | 107 | 63.47 | 69.48 |
| 24 | 94 | 36 | 63 | 72.98 | 73.44 | 48 | 41 | 40 | 71 | 75.97 | 80.70 |
| 24 | 99 | 54 | 83 | 91.88 | 90.89 | 48 | 32 | 44 | 82 | 82.09 | 86.53 |
| 6 | 45 | 14 | 47 | 66.59 | 65.14 | 48 | 84 | 46 | 57 | 74.45 | 78.60 |
| 6 | 55 | 16 | 25 | 66.90 | 65.36 | 48 | 59 | 55 | 80 | 89.10 | 92.57 |
| 6 | 6 | 18 | 41 | 78.38 | 76.87 | 6 | 54 | 16 | 86 | 67.09 | 65.55 |
| 6 | 32 | 19 | 69 | 74.56 | 72.94 | 6 | 42 | 26 | 76 | 80.38 | 78.33 |
| 8 | 29 | 35 | 135 | 91.81 | 89.54 | 6 | 21 | 35 | 90 | 94.28 | 91.66 |
| 8 | 65 | 42 | 105 | 92.72 | 89.82 | 6 | 29 | 41 | 111 | 99.38 | 96.28 |
| 24 | 55 | 36 | 103 | 80.36 | 80.92 | 10 | 47 | 16 | 99 | 66.50 | 65.72 |
| 24 | 13 | 50 | 72 | 103.75 | 103.30 | 10 | 61 | 23 | 92 | 71.57 | 70.40 |
| 24 | 36 | 11 | 54 | 56.40 | 58.37 | 10 | 27 | 25 | 122 | 80.21 | 79.00 |
| 24 | 34 | 20 | 71 | 66.70 | 68.35 | 12 | 77 | 22 | 48 | 66.49 | 65.71 |
| 24 | 29 | 32 | 95 | 80.87 | 81.78 | 12 | 70 | 25 | 65 | 71.12 | 70.18 |
| 24 | 11 | 67 | 88 | 122.87 | 120.97 | 12 | 57 | 42 | 101 | 92.32 | 90.19 |
| 12 | 35 | 20 | 82 | 72.23 | 71.66 |  | | | | | |
| 12 | 30 | 20 | 88 | 73.18 | 72.62 |  | | | | | |
| 12 | 19 | 49 | 95 | 107.23 | 104.63 |  | | | | | |
| 12 | 25 | 52 | 84 | 109.40 | 106.54 |  | | | | | |
| 36 | 81 | 24 | 73 | 56.49 | 60.03 |  | | | | | |
| 36 | 46 | 25 | 53 | 64.21 | 67.78 |  | | | | | |
| 36 | 25 | 37 | 85 | 81.42 | 84.19 |  | | | | | |
| 36 | 59 | 52 | 75 | 91.52 | 93.02 |  | | | | | |
| 12 | 70 | 27 | 97 | 73.33 | 72.26 |  | | | | | |
| 12 | 19 | 45 | 102 | 102.82 | 100.54 |  | | | | | |
| 12 | 43 | 50 | 104 | 103.79 | 101.05 |  | | | | | |

PLE: Programming language experience (months), R: Reused code, N&C: New and changed code, E: Effort (minutes), MLR: Effort predicted from the multiple linear regression model, GP: Effort predicted from genetic programming model.
