# Supplementary material for: Genetic Programming as Alternative for Predicting Development Effort of Individual Software Projects
Source: PLoS One. 2012 Nov 30;7(11):e50531. doi: 10.1371/journal.pone.0050531 (PMC3511534; doi:10.1371/journal.pone.0050531)
Supplement: Appendix S2 — Data of projects for validating models. (DOC) [file pone.0050531.s002.doc]

**Appendix B. Data of projects for validating models.**

| PLE | R | N&C | E | MLR | GP | PLE | R | N&C | E | MLR | GP | PLE | R | N&C | E | MLR | GP |
| --- | --- | --- | --- | --- | --- | --- | --- | --- | --- | --- | --- | --- | --- | --- | --- | --- | --- |
| 12 | 6 | 11 | 68 | 67.80 | 67.62 | 18 | 54 | 20 | 59 | 65.77 | 66.27 | 24 | 29 | 33 | 92 | 81.98 | 82.81 |
| 12 | 16 | 19 | 67 | 74.73 | 74.25 | 18 | 21 | 31 | 71 | 84.15 | 84.03 | 24 | 52 | 43 | 127 | 88.65 | 88.67 |
| 12 | 9 | 50 | 134 | 110.23 | 107.56 | 18 | 61 | 34 | 86 | 79.88 | 79.46 | 24 | 44 | 65 | 93 | 114.42 | 112.62 |
| 6 | 100 | 20 | 102 | 62.79 | 60.96 | 18 | 66 | 50 | 93 | 96.58 | 94.89 | 12 | 52 | 20 | 95 | 69.01 | 68.40 |
| 6 | 100 | 35 | 86 | 79.33 | 76.51 | 36 | 42 | 10 | 83 | 48.43 | 52.63 | 12 | 35 | 30 | 116 | 83.26 | 82.07 |
| 6 | 101 | 70 | 108 | 117.73 | 112.02 | 36 | 47 | 16 | 45 | 54.10 | 58.14 | 12 | 28 | 47 | 85 | 103.32 | 100.86 |
| 12 | 46 | 9 | 59 | 58.02 | 57.76 | 36 | 25 | 27 | 83 | 70.39 | 73.88 | 36 | 66 | 40 | 56 | 76.97 | 79.41 |
| 12 | 9 | 30 | 87 | 88.18 | 87.05 | 36 | 30 | 38 | 121 | 81.57 | 84.26 | 36 | 43 | 62 | 81 | 105.57 | 106.26 |
| 12 | 21 | 30 | 75 | 85.91 | 84.75 | 36 | 52 | 21 | 64 | 58.67 | 62.45 | 36 | 37 | 62 | 125 | 106.71 | 107.41 |
| 12 | 46 | 42 | 75 | 94.41 | 92.30 | 36 | 30 | 25 | 56 | 67.24 | 70.84 | 12 | 39 | 28 | 54 | 80.29 | 79.23 |
| 12 | 40 | 49 | 109 | 103.26 | 100.60 | 36 | 52 | 44 | 69 | 84.02 | 86.19 | 12 | 8 | 39 | 98 | 98.29 | 96.51 |
| 12 | 47 | 52 | 77 | 105.24 | 102.32 | 36 | 50 | 53 | 111 | 94.33 | 95.76 | 12 | 32 | 43 | 99 | 98.16 | 96.00 |
| 12 | 30 | 53 | 148 | 109.56 | 106.60 | 12 | 43 | 11 | 80 | 60.80 | 60.53 | 12 | 23 | 46 | 155 | 103.17 | 100.80 |
| 8 | 58 | 24 | 68 | 74.20 | 72.60 | 12 | 33 | 22 | 63 | 74.82 | 74.14 | 12 | 44 | 22 | 64 | 72.73 | 72.03 |
| 8 | 55 | 46 | 75 | 99.02 | 95.83 | 12 | 37 | 34 | 113 | 87.29 | 85.81 | 12 | 32 | 40 | 70 | 94.85 | 92.93 |
| 8 | 37 | 37 | 93 | 92.50 | 90.06 | 36 | 53 | 35 | 48 | 73.91 | 76.77 | 12 | 29 | 50 | 78 | 106.44 | 103.73 |
| 8 | 18 | 78 | 104 | 141.30 | 135.45 | 36 | 23 | 43 | 52 | 88.41 | 90.73 | 12 | 18 | 48 | 74 | 106.32 | 103.80 |
| 36 | 40 | 49 | 116 | 91.81 | 93.60 | 36 | 17 | 40 | 90 | 86.24 | 88.81 | 24 | 21 | 12 | 39 | 60.34 | 62.34 |
| 36 | 66 | 56 | 58 | 94.61 | 95.75 | 36 | 6 | 52 | 57 | 101.55 | 103.18 | 24 | 17 | 25 | 28 | 75.43 | 76.84 |
| 36 | 17 | 56 | 103 | 103.88 | 105.14 | 60 | 31 | 20 | 64 | 50.09 | 58.43 | 24 | 36 | 38 | 89 | 86.16 | 86.61 |
| 36 | 13 | 57 | 87 | 105.74 | 106.93 | 60 | 36 | 32 | 60 | 62.37 | 69.94 | 60 | 87 | 29 | 50 | 49.41 | 57.07 |
| 7 | 35 | 59 | 129 | 117.61 | 113.20 | 60 | 51 | 47 | 112 | 76.07 | 82.45 | 60 | 55 | 42 | 62 | 69.80 | 76.57 |
| 7 | 32 | 29 | 74 | 85.11 | 83.07 | 60 | 56 | 55 | 72 | 83.95 | 89.65 | 60 | 63 | 61 | 68 | 89.24 | 94.41 |
| 7 | 22 | 27 | 110 | 84.80 | 82.91 | 48 | 49 | 17 | 68 | 49.10 | 55.32 | 60 | 76 | 80 | 97 | 107.72 | 111.19 |
| 7 | 20 | 27 | 108 | 85.17 | 83.30 | 48 | 28 | 47 | 129 | 86.15 | 90.36 | 6 | 64 | 35 | 63 | 86.14 | 83.41 |
| 6 | 50 | 19 | 70 | 71.15 | 69.49 | 18 | 43 | 28 | 67 | 76.68 | 76.72 | 6 | 58 | 65 | 71 | 120.35 | 115.18 |
| 6 | 46 | 26 | 51 | 79.63 | 77.57 | 18 | 42 | 34 | 93 | 83.48 | 83.10 | 6 | 47 | 44 | 76 | 99.28 | 95.90 |
| 6 | 13 | 56 | 88 | 118.95 | 114.66 | 18 | 27 | 30 | 70 | 81.91 | 81.85 | 6 | 34 | 64 | 94 | 123.79 | 118.77 |
| 6 | 12 | 48 | 99 | 110.32 | 106.70 | 18 | 10 | 45 | 79 | 101.66 | 100.52 | 36 | 69 | 16 | 65 | 49.94 | 53.92 |
| 36 | 35 | 18 | 77 | 58.58 | 62.56 | 60 | 13 | 48 | 139 | 84.37 | 90.75 | 36 | 92 | 23 | 69 | 53.30 | 56.88 |
| 36 | 14 | 27 | 79 | 72.47 | 75.99 | 60 | 22 | 69 | 132 | 105.82 | 110.39 | 36 | 93 | 52 | 74 | 85.09 | 86.50 |
| 36 | 30 | 54 | 100 | 99.21 | 100.61 | 24 | 15 | 17 | 81 | 66.98 | 68.83 | 6 | 59 | 17 | 57 | 67.24 | 65.65 |
| 36 | 30 | 57 | 99 | 102.52 | 103.67 | 24 | 11 | 33 | 63 | 85.38 | 86.26 | 6 | 27 | 20 | 64 | 76.61 | 74.95 |
| 60 | 41 | 15 | 42 | 42.68 | 51.22 | 24 | 23 | 59 | 167 | 111.78 | 110.54 | 6 | 21 | 21 | 113 | 78.85 | 77.15 |
| 60 | 22 | 20 | 49 | 51.79 | 60.15 | 24 | 35 | 19 | 93 | 65.40 | 67.11 | 4 | 26 | 20 | 74 | 77.75 | 75.72 |
| 60 | 34 | 26 | 57 | 56.14 | 64.12 | 24 | 44 | 27 | 77 | 72.52 | 73.74 | 4 | 34 | 28 | 69 | 85.06 | 82.53 |
| 60 | 34 | 51 | 60 | 83.70 | 89.79 | 24 | 39 | 38 | 100 | 85.59 | 86.04 | 4 | 62 | 36 | 82 | 88.58 | 85.41 |
| 12 | 24 | 26 | 67 | 80.93 | 80.03 | 24 | 49 | 65 | 128 | 113.47 | 111.66 | 4 | 45 | 50 | 151 | 107.23 | 103.00 |
| 12 | 7 | 37 | 63 | 96.27 | 94.65 | 12 | 69 | 27 | 51 | 73.51 | 72.45 |  | | | | | |
| 36 | 48 | 15 | 62 | 52.81 | 56.88 | 12 | 39 | 34 | 69 | 86.91 | 85.43 |  | | | | | |
| 36 | 27 | 20 | 63 | 62.30 | 66.20 | 12 | 76 | 57 | 93 | 105.26 | 101.85 |  | | | | | |
| 36 | 64 | 35 | 67 | 71.83 | 74.66 | 12 | 58 | 67 | 132 | 119.70 | 115.46 |  | | | | | |
| 36 | 40 | 54 | 130 | 97.32 | 98.70 | 10 | 86 | 11 | 56 | 53.61 | 52.87 |  | | | | | |
| 6 | 38 | 17 | 95 | 71.22 | 69.68 | 10 | 65 | 17 | 45 | 64.20 | 63.33 |  | | | | | |
| 6 | 23 | 64 | 110 | 125.88 | 120.88 | 10 | 59 | 11 | 31 | 58.72 | 58.05 |  | | | | | |
| 6 | 10 | 48 | 128 | 110.70 | 107.08 | 10 | 51 | 12 | 40 | 61.34 | 60.67 |  | | | | | |

PLE: Programming language experience (months), R: Reused code, N&C: New and changed code, E: Effort (minutes), MLR: Effort predicted from the multiple linear regression model, GP: Effort predicted from the genetic programming model.
